# Customized allogeneic bone grafts for maxillary horizontal augmentation: A 5‐year follow‐up radiographic and histologic evaluation

**DOI:** 10.1002/ccr3.2777

**Published:** 2020-03-11

**Authors:** Frank Rudolf Kloss, Vincent Offermanns, Phil Donkiewicz, Anita Kloss‐Brandstätter

**Affiliations:** ^1^ Private Clinic for Oral- and Maxillofacial Surgery Lienz Austria; ^2^ Department of Cranio‐, Maxillofacial and Oral Surgery Medical University Innsbruck Innsbruck Austria; ^3^ Department of Oral Surgery and Dental Emergency Care Faculty of Health School of Dentistry Witten/Herdecke University Witten North Rhine‐Westphalia Germany; ^4^ Carinthia University of Applied Sciences Villach Austria

**Keywords:** alveolar ridge augmentation, case report, dental implants, graft remodeling, individualized freeze‐dried bone allograft

## Abstract

We report the histological evaluation of an individualized allogeneic bone block 5 years after alveolar ridge augmentation. The biopsy showed a well‐vascularized lamellar bone with fatty incorporations without any avital allograft remnants. The presence of osteocytes, lining cells, macrophages, and blood vessels indicated a healthy and vital bone tissue.

## Graphical abstract

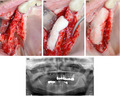

## 1 INTRODUCTION

Customized allogeneic bone grafts have been demonstrated to function as an ideal scaffold for the augmentation of alveolar ridge defects with high success rates and great predictability.^1^, ^2^, ^3^, ^4^, ^5^ Compared to other biomaterials, allografts provide favorable characteristics in terms of graft and particle size as well as a favorable inter‐ and intraparticulate porous geometry to supply osteoconductive effects and to maintain the graft volume.^6^ Remarkably, it has recently been reported that in terms of implant survival rates and volume stability spongious freeze‐dried allogeneic bone blocks are not inferior to monocortical autogenous bone blocks.^7^

Nowadays, computer‐aided design/computer‐aided manufacturing (CAD/CAM) technology enables the individualized manufacturing of allogeneic bone blocks for complex alveolar ridge augmentation procedures. Several case reports already demonstrated the high precision fit and the successful application of customized allograft blocks.^2^, ^3^, ^4^, ^5^ However, so far there are limited reports about the long‐term fate of the implanted bone allografts.^8^, ^9^, ^10^ With this case report, we were able to evaluate the remodeling of a customized allograft by taking a biopsy in a patient five years after alveolar ridge augmentation.

To the best of our knowledge, this is the first case report with a follow‐up period of 5 years on the clinical performance of a customized freeze‐dried cancellous allogeneic bone block with histological evaluation.

## 2 MATERIALS AND METHODS

### 2.1 Overview of the clinical case

This is a retrospective case report, completed according to the CARE guidelines. In 2013, a 66‐year‐old patient presented with a massive horizontal atrophy of the right maxillary alveolar ridge (residual width: 2‐3 mm). This spacious osseous defect was treated with a customized freeze‐dried allogeneic bone graft. Three titanium implants were inserted 6 months after augmentation. In 2019, a biopsy was taken during a vestibuloplasty and analyzed histologically with toluidine blue. The patient gave informed consent to taking a bone biopsy for histological evaluation.

### 2.2 Patient

**Figure 1:**
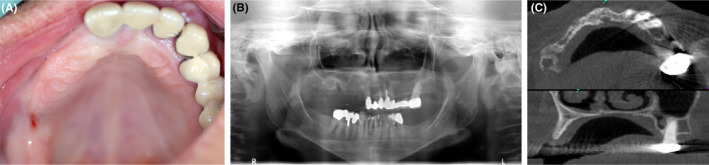
Preoperative intraoral status of a 66‐y‐old female patient with a type II bone defect in the maxilla. A, Palatal view of the defect area; B, panoramic radiograph of the patient demonstrated massive bone loss in the right maxilla; C, axial (upper panel) and sagittal (lower panel) section of the CBCT illustrating the region of interest

In 2013, a 66‐year‐old female patient presented with the wish for a fixed prosthetic rehabilitation in site 12‐17 of the maxilla. Preliminary radiological evaluations showed a substantial horizontal atrophy of the alveolar ridge with a residual ridge width of 2‐3 mm, resembling a type II defect according to Chen & Buser^11^ (Figure 1) or a Class 4 defect (horizontal ridge defect requiring bone augmentation before implant placement) according to Benic & Hämmerle.^12^ The patient exhibited a treated and stable periodontitis with current gingival health according to Chappel et al^13^ and was in regular recall. The bony situation at the teeth proved to be stable. There was no bleeding on probing. The patient suffered from a mild systemic disease (ASA 2, according to the physical status classification system). The safety assessment code matrix for the patient according to Dawson and Chen^14^ can be found in Table 1.

**Table 1:** Safety assessment code matrix of the patient

	Low risk	Medium risk	High risk
Health status	Healthy, cooperative, and without immunological restriction		
Tobacco use	Nonsmoker	1‐10/d	>10/d
Patient's claim	Low	Medium	High
Height of the smile line	Low	Medium	High
Gingival biotype	fabric strong, flat garland shape	Medium strong, average garland shape	Fabric weak, steep garland shape
Dental form type	Rectangular		Triangular
Local infection	None	Chronic	Acute
Bone level at neighboring teeth	≤ 5 mm from the contact point	5.5‐6.5 mm from the contact point	≥ 7 mm from the contact point
Restoration status of neighboring teeth	Untouched		Restored
Width of the gap	1 tooth (≥7 mm)	1 tooth (≤7 mm)	≥2 teeth
Soft tissue condition	Intact	Reduced	Defective
Bone volume	No defect	Horizontal defect	Vertical defect

If the patient was at low risk for one of the risk factors, the corresponding attribution was highlighted in green color. If the patient was at medium risk for one of the risk factors, the corresponding attribution was highlighted in orange color. If the patient was at high risk for one of the risk factors, the corresponding attribution was highlighted in red color.

### 2.3 Custom‐milled freeze‐dried allogeneic bone graft

Our treatment plan for this roomy osseous defect was a guided bone regeneration carried out with a custom‐milled freeze‐dried allogeneic bone graft (maxgraft^®^ bonebuilder, botiss biomaterials GmbH). A CBCT scan with a layer thickness of 0.3 mm of the jaw area was used to virtually design the individualized allograft based on a three‐dimensional reconstruction of the alveolar ridge defect using the Freeform software (Geomagic). To this end, a DICOM data set derived from a CBCT scan was uploaded onto the manufacturer's website. A technician then produced a digital model of the patient's jaw from that data set and virtually designed the missing bone fragment. The design of this fragment was then uploaded into a 3D‐milling unit in which a wet‐chemically processed freeze‐dried bone block was placed. The individualized allogeneic bone block was shaped from a chemically processed and lyophilized cancellous bone of a femoral head derived from a living donor (Allotec^®^ process, Cells + Tissue Bank Austria, Krems, Austria^15^). The bone was derived from living donors undergoing hip arthroplasty, washed in an ultrasonic bath, immersed in ethanol, diethyl ether, and hydrogen peroxide, and freeze‐dried in order to remove cells and noncollagen proteins. After the milling process, the customized bone block was wrapped into two blisters and sterilized using gamma irradiation.

### 2.4 Surgical procedure

The preoperative clinical examinations, the overall surgical procedure, and the postoperative care followed our previous description of alveolar ridge augmentation.^7^

**Figure 2:**
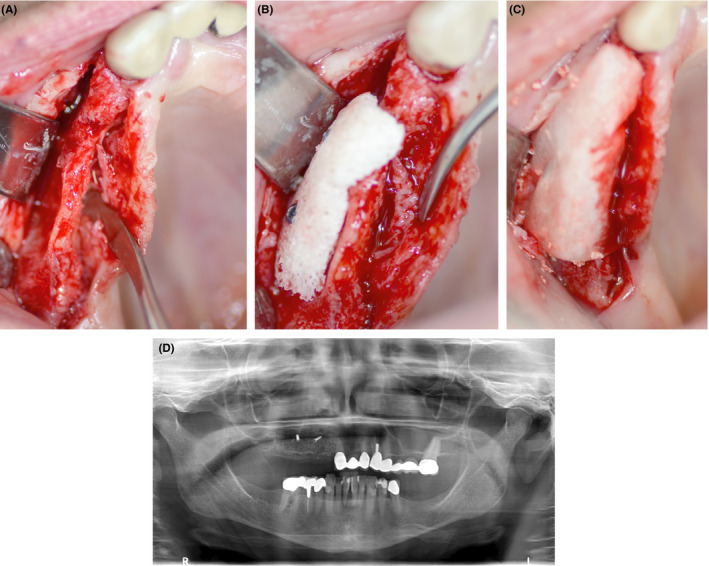
Intraoperative clinical situation during augmentation. A, After preparation of the defect area; B, insertion of the customized bone allograft into the defect area; C, intraoperative situation after additional application of bovine bone particles and a pericard membrane; D, panoramic radiograph after augmentation

In short, the native bone was punctured with drills to guarantee vascularization of the allograft (Figure 2). The customized allograft was obtained sterile from the double blister. The block exactly matched the defect's geometry and was firmly secured on the maxillary ridge with two 1.5 mm osteosynthesis screws (Komet), which also promoted gentle rehydration of the block with the patient's blood. The small void spaces were stuffed with a bovine bone substitute material (Endobon^®^, Biomet 3i LLC). The surgical site was protected with a resorbable barrier membrane made from porcine pericardium (Jason^®^ Membrane, Botiss Biomaterials GmbH). The membrane was not fixed with pins or suture; instead, it was placed palatally under the periosteum and vestibular over the bone block. The intervention was carried out under local anesthesia in our private practice in Lienz, Austria.

Routine postoperative care included administration of amoxicillin and clavulanic acid (625 mg, administered orally, three times a day for 4 days), ibuprofen (600 mg, administered orally, every 6 hours as needed), and mouthwashes (0.2% chlorhexidine, three times daily for 7 days). The patient was recalled at monthly intervals for a period of 6 months to detect possible complications, such as infection, pain, discomfort, graft exposure, and graft mobility.

### 2.5 Implantation

**Figure 3:**
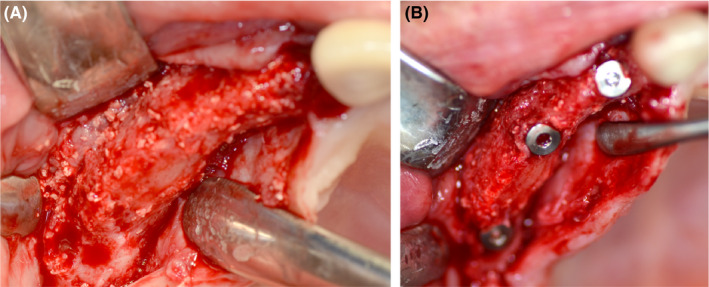
Intraoperative clinical situation after 6 mo at re‐entry. A, The allogeneic bone block was well integrated and a cortical layer was established. Remnants of the bovine bone particles are visible. The grafted area showed sufficient bone volume and vital tissue for implant placement six months after augmentation. B, After implantation regio 12, 14, and 16

**Figure 4:**
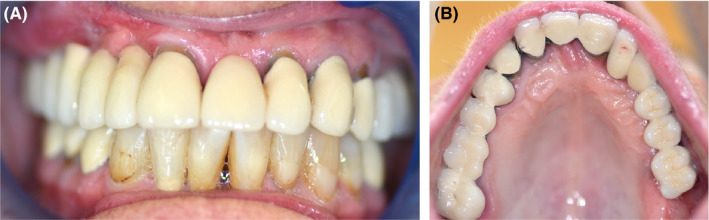
Status after prosthetic restoration of the patient 3 y after augmentation. A, Frontal view; B, palatal view; C, panoramic radiograph showing good integration of the allograft and the implants

The implants were placed after 6 months of healing as described previously.^7^ Fixation screws were removed, and the graft stability was assessed. The customized allograft was well integrated and a cortical bone layer was established on the surface of the new formed bone tissue. The allograft presented vital tissue and satisfactory bone volume for stable implantation. The patient received three titanium implants in sites 12, 14, and 16 (blueSKY; bredent medical GmbH & Co. KG) (Figure 3). The grafted bone stayed steady during drilling and implant placement, without graft separation. In order to enhance soft tissue thickness and maintain an esthetic appearance, a vestibuloplasty was carried out using a porcine collagen matrix (Mucoderm^®^, Botiss). Three months after implantation, the implants were uncovered and the patient received a fixed partial denture (Figure 4).

### 2.6 Histological examination

After 5 years, no new vestibuloplasty was required. However, a bony “spur” disturbed the patient. This “spur” turned out to be bovine granules, which were removed. In the course of this small surgery, a bone core biopsy was retrieved from the area between site 12 and 14 and processed for histologic examination. The main procedure of histologic examination follows our previous description.^16^

The following parameters were histologically examined: integration pattern of the graft, fibrosis, hemorrhage, necrosis, vascularization and the presence of neutrophils, lymphocytes, plasma cells, macrophages, and multinucleated giant cells.^16^

### 2.7 Radiographic analyses

**Figure 5:**
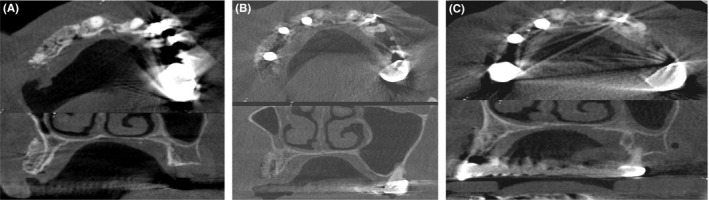
Cone beam computed tomography scans (upper panel: axial section; lower panel: sagittal section). A, directly post augmentation; B, 6 mo post augmentation; C, 5 y postaugmentation

The radiographic analyses followed the procedure described before.^7^ Four CBCT scans were taken: one before treatment (Figure 1), one directly after augmentation, one after 6 months of healing, and one after 5 years (Figure 5).

## 3 RESULTS

CAD/CAM manufactured allogeneic bone blocks represent an innovative approach for alveolar ridge augmentation, as the customized allograft exactly matches the contour of the defect and thus enables restoration of extensive osseous defects. This case report demonstrates the histological and radiographic results of a customized allograft 5 years after augmentation.

The patient was followed up for more than 5 years. There were no signs of infection, wound dehiscence, block graft exposure, or other postoperative complications during the healing period following bone augmentation. At the time of implantation, the customized allogeneic bone block was well integrated into the recipient site. All implants were stable during follow‐up.

A stable prosthesis hold was found both after 3 months and after 5 years. No bleeding on probing was recognizable at six measuring points per implant. After 5 years, there was a slight recession of the peri‐implant mucosa, but this did not lead to the exposure of parts of the implant (approx. 0.5‐1 mm decrease in peri‐implant mucosa on the implant crown). There was no evidence of mucositis or even peri‐implantitis.

**Figure 6:**
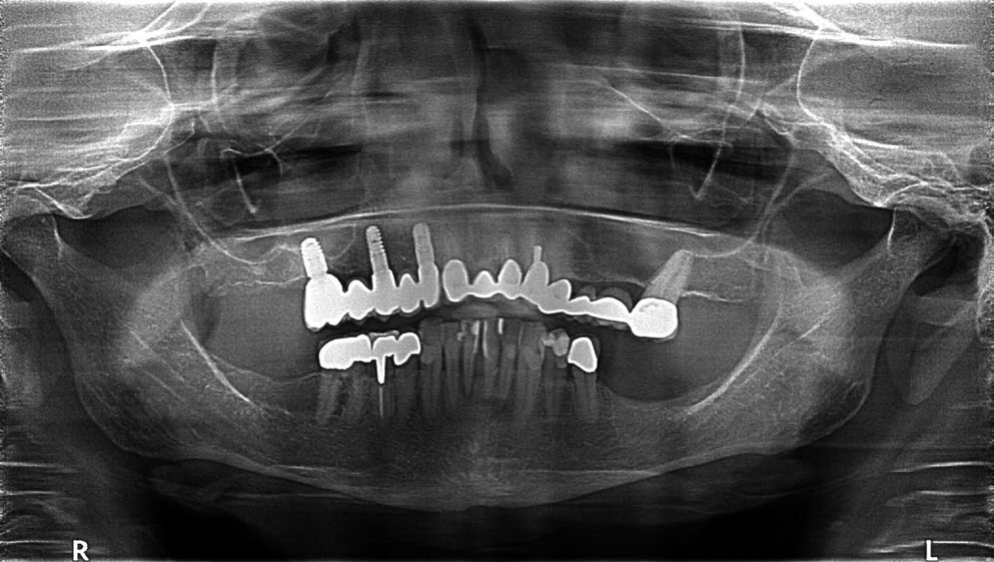
Panoramic radiograph 5 y after augmentation showing good integration of the allograft and the implants with minimal loss of bone at the implant shoulder

The panoramic radiograph 3 years after implantation showed stable implant conditions with minimal bone loss on the implant shoulder (Figure 4). The panoramic radiograph after 5 years showed the same implant and bone conditions as the radiography 2 years before, with minimal bone loss on the implant shoulder (Figure 6).

**Figure 7:**
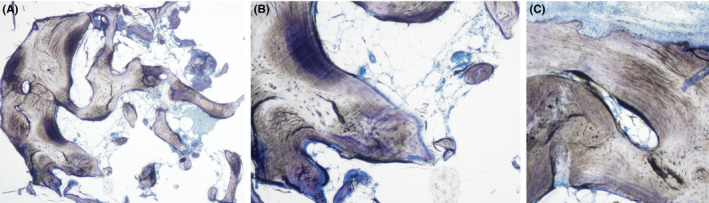
Histological result 5 y after augmentation. A, The overview shows a well‐vascularized (black arrows mark blood vessels) lamellar bone with fatty incorporations (yellow asterisks). No avital remnants of the allogeneic bone block are visible. B, Throughout the entire specimen, osteoblastic lining cells (white arrowheads) form a new layer of unmineralized osteoid adjacent to the mineralized bone substance. C, Vital osteocytes are embedded within the mineralized bone substance (10× magnification) (black arrowheads) and Howship's lacunae with adjacent osteoclasts represent ongoing bone remodeling (gray arrowheads)

The histological specimens indicated vital bone tissue with greasy inclusions, which is characteristic for the maxilla (Figure 7). The 10‐fold magnification showed vital osteocytes around Haversian canals demonstrating intact osteons. Several cut vessels were found throughout the histology, demonstrating successful vascularization and vital tissue. The presence of osteoclasts in Howship's lacunae emphasizes ongoing remodeling processes. Furthermore, margins of light blue color with lining cells producing the unmineralized osteoid were found on the surface of the bone. No avital remnants of the allograft were detectable, while the presence of osteocytes, lining cells, macrophages, and blood vessels demonstrated all hallmarks of healthy and vital bone tissue. In general, the analyzed bone tissue in the augmented region reflected a well‐vascularized lamellar bone with fatty incorporations, which is typical for the maxilla, and exhibited no differences from healthy native bone tissue.

## 4 DISCUSSION

Although satisfactory results can be achieved with grafting of autologous bone blocks, this method is accompanied by several shortcomings, such as extended surgery time, restricted graft acquisition, risk for impairment of the neighboring teeth, neurosensory deficits, donor area flap exposure, bleeding, and infection.^7^, ^17^, ^18^ Due to these drawbacks, other grafting materials are required that show lower or none morbidity and effortless appliance. For their application in dental medicine, allogeneic bone blocks were shown to be competent of overcoming many of the weaknesses of autogenous block grafting, particularly difficulties related to the donor site.^17^, ^19^

A further improvement is the customization of allogeneic bone blocks with a precise defect fit, which is especially of added value for complex bone augmentations, as the space between residual bone and bone graft can be condensed to a minimum. Thereby, the physical contact between graft and recipient site is enhanced, thus allowing optimal trophic support and revascularization of the allograft through integration/replacement (creeping substitution) at the recipient site.^20^, ^21^ Additionally, this uninterrupted connection to the adjacent bone tissue enables a rapid bony integration. Moreover, the utilization of custom‐milled allogeneic bone blocks reduces the operation time, as shaping of the block is obsolete.

In a recent consensus statement, Sanz et al summarized that allogenic bone replacement grafts provided similar mechanical properties as the autologous bone and they might contain the collagenous matrix and proteins of natural bone, although they lacked viable cells.^22^ In addition, Sanz et al outlined that the handling properties were comparable to autologous bone, although the reduced surgical time needed for their implantation, in addition to their increased availability were clear advantages of allografts when compared with autologous bone.^22^ Possible disease transmission, potential unwanted immune reactions, variation in resorption rates, and a possible impairment to achieve vascularization of the grafted site were reported as disadvantages.^22^

In 2018, we compared the rates of resorption between autogenous and allogenic bone blocks for alveolar ridge augmentation. For both methods, 21 patients, who had only a single tooth gap and who received an alveolar ridge augmentation before implantation with a single implant, were retrospectively evaluated using radiographics. The allogenic bone blocks used were small standard blocks and were customized to the defect size during the course of the operation. We were able to show that, contrary to previous claims, there were no differences in the resorption rates between autogenous and allogenic bone blocks within an observation period of 12 months.^7^

Although regeneration of large osseous defects has been demonstrated with a variety of augmentation techniques, most require bone harvesting procedures. One example is the application of a mixture of particulate autologous bone and anorganic bovine bone in combination with collagen membranes in a conventional GBR approach, with which 5 mm horizontal bone gain was achieved.^23^ However, biopsy specimens of sinuses grafted with xenogenic bone grafts demonstrated that this material is rather integrated than remodeled, which results in less vital bone being regenerated.^24^

We decided to use the allogenic bone block for and particulate xenogenic bone because of its volume stability in order to minimize graft resorption, as previous reports have demonstrated.^25^ Additionally, a collagen barrier membrane was applied to stabilize the bovine granular material and minimize graft resorption. Collagen membranes were shown to delay the mineralization process of bone grafts while substantially increasing the volume stability of allogenic bone grafts.^26^

Several studies have already demonstrated that autologous and allogenic bone blocks show comparable vital bone formation as well as comparable results in implant success rates.^27^ Although recent reports have demonstrated remodeling capacities of collagen‐containing xenogenic bone blocks in horizontal ridge augmentation procedures, some drawbacks regarding the clinical results of this augmentation procedure have been reported.^28^, ^29^

Another technique, which might become more relevant in the future, is the application of alloplastic CAD/CAM manufactured bone blocks; however, there are little reports about the clinical performance and long‐term results with biopsy specimens are missing.^30^

In the present case report, we present a single patient with a very large defect in the alveolar ridge. For this patient, a customized bone block was milled from a donor femoral head, which was then fixed with osteosynthesis screws. Three implants were placed in this personalized allogeneic bone block. The observation period was 5 years. We could show with histological analysis that the donor bone from the hip had almost completely been transformed into maxillary bone. This result is an absolute novelty, because so far there was no case report on personalized allogeneic bone blocks, which on the one hand had such a long observation period and on the other hand had such a well‐founded histological work‐up.

One intriguing aspect of this case report is that other procedures facilitating the regeneration of such an extensive bone defect, like autologous bone blocks or individualized titan meshes, depend on harvesting autologous bone tissue which was hard to conduct within the oral cavity of the here described patient.^23^, ^31^ As the residual width of the ridge was insufficient for ridge splitting, one very prominent option for the augmentation of the patient's bone defect is the application of bone harvested from the iliac crest, which would have meant surgery under general anesthesia in a university hospital. In the mountainous and remote alpine district of Lienz, this would not have been feasible for the patient. Therefore, we also wanted to demonstrate the applicability of such a surgery even in private practices under local anesthesia.

To the best of our knowledge, our case report is the first to report a long‐term observation of the remodeling behavior of a customized allogeneic bone block for alveolar ridge augmentation over a period of more than 5 years. This case report showed the complete remodeling of the customized allogeneic block with the formation of new vital bone, which was indistinguishable from healthy native bone tissue. Therefore, our study together with results published by others emphasizes customized allogeneic bone blocks to demonstrate a successful and lasting treatment concept for augmentation of complex defects of the alveolar ridge.^4^, ^32^, ^33^

## CONFLICT OF INTEREST

None declared.

## AUTHOR CONTRIBUTIONS

FRK: treated the patient; designed the customized allograft; performed surgical procedures; was involved in drafting the manuscript; and gave final approval of the version to be published. VO: was involved in drafting the manuscript; performed histological analyses; and gave final approval of the version to be published. PD: was involved in drafting the manuscript; made substantial contributions to conception and design; and gave final approval of the version to be published. AK‐B: was involved in drafting the manuscript; performed literature research; made substantial contributions to conception and design; and gave final approval of the version to be published.
